# The First Occurrence in the Fossil Record of an Aquatic Avian Twig-Nest with Phoenicopteriformes Eggs: Evolutionary Implications

**DOI:** 10.1371/journal.pone.0046972

**Published:** 2012-10-17

**Authors:** Gerald Grellet-Tinner, Xabier Murelaga, Juan C. Larrasoaña, Luis F. Silveira, Maitane Olivares, Luis A. Ortega, Patrick W. Trimby, Ana Pascual

**Affiliations:** 1 CONICET at CRILAR, Anillaco, Argentina; 2 The Field Museum, Chicago, Illinois, United States of America; 3 The Journey Museum, Rapid City, South Dakota, United States of America; 4 Departamento de Estratigrafía y Paleontología, Facultad de Ciencia y Tecnología, Euskal Herriko Unibertsitatea, Bilbao, Spain; 5 Instituto Geológico y Minero de España, Unidad de Zaragoza, Zaragoza, Spain; 6 Seção de Aves, Museu de Zoologia da Universidade de São Paulo, São Paulo, Brazil; 7 Australian Centre for Microscopy and Microanalysis, The University of Sydney, Sydney, Australia; The Pennsylvania State University, United States of America

## Abstract

**Background:**

We describe the first occurrence in the fossil record of an aquatic avian twig-nest with five eggs *in situ* (Early Miocene Tudela Formation, Ebro Basin, Spain). Extensive outcrops of this formation reveal autochthonous avian osteological and oological fossils that represent a single taxon identified as a basal phoenicopterid. Although the eggshell structure is definitively phoenicopterid, the characteristics of both the nest and the eggs are similar to those of modern grebes. These observations allow us to address the origin of the disparities between the sister taxa Podicipedidae and Phoenicopteridae crown clades, and traces the evolution of the nesting and reproductive environments for phoenicopteriforms.

**Methodology/Principal Findings:**

Multi-disciplinary analyses performed on fossilized vegetation and eggshells from the eggs in the nest and its embedding sediments indicate that this new phoenicopterid thrived under a semi-arid climate in an oligohaline (seasonally mesohaline) shallow endorheic lacustine environment. High-end microcharacterizations including SEM, TEM, and EBSD techniques were pivotal to identifying these phoenicopterid eggshells. Anatomical comparisons of the fossil bones with those of Phoenicopteriformes and Podicipediformes crown clades and extinct palaelodids confirm that this avian fossil assemblage belongs to a new and basal phoenicopterid.

**Conclusions/Significance:**

Although the Podicipediformes-Phoenicopteriformes sister group relationship is now well supported, flamingos and grebes exhibit feeding, reproductive, and nesting strategies that diverge significantly. Our multi-disciplinary study is the first to reveal that the phoenicopteriform reproductive behaviour, nesting ecology and nest characteristics derived from grebe-like type strategies to reach the extremely specialized conditions observed in modern flamingo crown groups. Furthermore, our study enables us to map ecological and reproductive characters on the Phoenicopteriformes evolutionary lineage. Our results demonstrate that the nesting paleoenvironments of flamingos were closely linked to the unique ecology of this locality, which is a direct result of special climatic (high evaporitic regime) and geological (fault system) conditions.

## Introduction

Avian fossil nests made from twigs are practically unknown in the fossil record as their fragile nature makes their survival a rare event. We report the first occurrence in the fossil record of such bird nest. This nest, with five eggs *in situ* was discovered in an Early Miocene lacustrine limestone bed in the Tudela Formation (Ebro Basin, Spain). The nest consists of cf. Fabaceae twigs and leaves and is interpreted to have been floating a few centimetres from the bottom of a sub-oxic and oligohaline endorheic lake before fossilization. Extensive examinations of the outcrops of the Tudela formation in the Ebro Basin reveal an autochthonous avian osteological and oological fossil assemblage that presently represents only a single taxon identified, hereby, as a new fossil phoenicopterid (paleoflamingo). This identification is confirmed by the characters of the exquisitely preserved eggshell of the five eggs in the nest.

The osteological features of this new species differ from all known and well-represented paleoflamingos and their allies that constitute an abundant widespread group of fossil birds in Europe [1]. Although high-end microcharacterization of eggshell fragments from the eggs in the nest clearly supports this flamingo identification, the nest style, the number of eggs and their size are similar to those of modern grebes. Considering the significantly divergent feeding, reproductive and nesting strategies between the now-well supported sister group Podicipedidae and Phoenicopteridae crown clades, coupled with the present lack of information in Palaelodidae, this unique fossil assemblage will have significant phylogenetic, biological and ecological implications for our understanding of the evolution of the flamingo lineage.

## Results

### Locality and horizon

The Ebro Basin is a triangular-shaped endorheic depression formed during the latest Eocene to the late Miocene in the foreland of the Pyrenees, the Iberian Range and the Catalan Coastal Ranges fold-and-thrust belts. This depression was occupied by a central lacustrine system, where alluvial and fluvial systems sourced in the surrounding mountain belts converged through a palustrine transitional zone [2]. The Tudela Formation is 655 meters thick and consists of distal alluvial, fluvial, palustrine and lacustrine sediments that accumulated in the western part of the central Ebro Basin during the Early and Middle Miocene [3]. These sediments crop out extensively at the Bardenas Reales de Navarra Natural Park in the vicinity of Tudela. Although sediments from the Tudela Formation are flat-lying throughout most of the Natural Park, tectonic deformation is apparent in the form of two families of joints and the occurrence of some reverse, strike-slip and normal faults with small offsets, rarely exceeding 2 meters [4].

Sediments from the Tudela Formation are grouped into 5 units [3] and show sedimentary facies identical to those described elsewhere in the central Ebro Basin (see [5]). Brown and red mudstones with interbedded sandstones indicate the development of distal fluvial and alluvial muddy flood plains at the foothills of the surrounding mountain belts, and are best represented in units 1 and 4. Thick (>2 m) limestone beds and associated grey marls are a result of the lacustrine system that developed in the central sector of the basin, mainly during accumulation of units 2 and 5. Ochre, yellow, pink and grey mudstones and thin (<1 m) limestone beds typify palustrine environments developed at intermediate positions, and are best represented in unit 3. Gypsum beds, which appear occasionally in unit 1 and also at a 7 meter-thick level (the so-called Fustiñana Gypsum) within unit 3, attest to deposition in saline mud flats.

The Tudela Formation grades laterally towards the east to the Zaragoza Gypsum Formation, which is composed of gypsum, glauberite, halite and anhydrite layers interbedded with grey mudstones and marls [6]. These sediments accumulated in a shallow perennial saline lake system that occupied the center of the Ebro basin during the Lower Miocene. In view of the lack of any physical barrier separating the fresh-water and saline lake systems of the Tudela and Zaragoza Gypsum formations, Arenas & Pardo [5] have proposed the existence of a single lake system in which climatically-controlled lake-level variations resulted in the facies arrangement of these two formations. Thus, wetter periods witnessed the occurrence of a single body of diluted water with extensive palustrine margins, leading to simultaneous accumulation of grey marls and mudstones in the centre of the basin (Zaragoza Gypsum Formation) and of lacustrine/palustrine carbonates and mudstones in the lake margins (Tudela Formation). Drier periods induced lower lake levels and caused sulfate depositional environments in the center of the basin (evaporites of the Zaragoza Gypsum Formation) and to progradation of distal alluvial facies towards the center of the lake system (red-brown-yellow mudstones and sandstones of the Tudela Formation). This model is supported by sedimentological evidence for the influence of orbital cycles in lacustrine [7] and distal alluvial [8] sedimentation in the Ebro basin during the Early Miocene, with precession and eccentricity modulating the facies arrangement. This pattern of sedimentation prevailed until the beginning of the Middle Miocene, when overall wetter conditions linked to the Mid-Miocene climate optimum led to the development of a permanent fresh-water lake and to the accumulation of lacustrine limestones and marls throughout the central part of the basin (unit 5 of the Tudela Formation and Alcubierre Formation).

A detailed paleontological survey of the Bardenas Reales has yielded 16 fossil localities, all within palustrine-lacustrine grey marls, distributed throughout the Tudela Formation. This ongoing survey has resulted in the recovery of fossil remains of a wide variety of vertebrates such as reptiles (lizards, amphisbaenians, snakes, turtles, and crocodilians [9], [10]), amphibians (anurans and urodeles [9]), bony fish (Cypriniformes [9]), birds [9], and mammals (insectivores, chiropters rodents, lagomorphs, artiodactyls and perissodactyls [9], [11], [12], [13]). Turtles, crocodiles, amphibians, and fish are autochthonous lacustrine-palustrine faunal components. The continental fossils were autochthonous but transported to the palustrine-lacustrine areas by streams [9]. These faunal assemblages support the inference of a subtropical climate in the Ebro Basin throughout the Early Miocene and the beginning of the Middle Miocene [9], [11]. Magnetostratigraphic data [3] indicate that sediments from the Tudela Formation range between 20.1 and 15.5 Ma according to the astronomically-tuned time scale (ATNTS2004) of Lourens et al. [14], thereby spanning the Miocene climatic optimum [15]. Avian eggshell fragments have been found in all the studied fossil localities in the concentrated residue that resulted from washing and sieving sediment in search of small mammal teeth and vertebrate remains.

**Figure 1:**
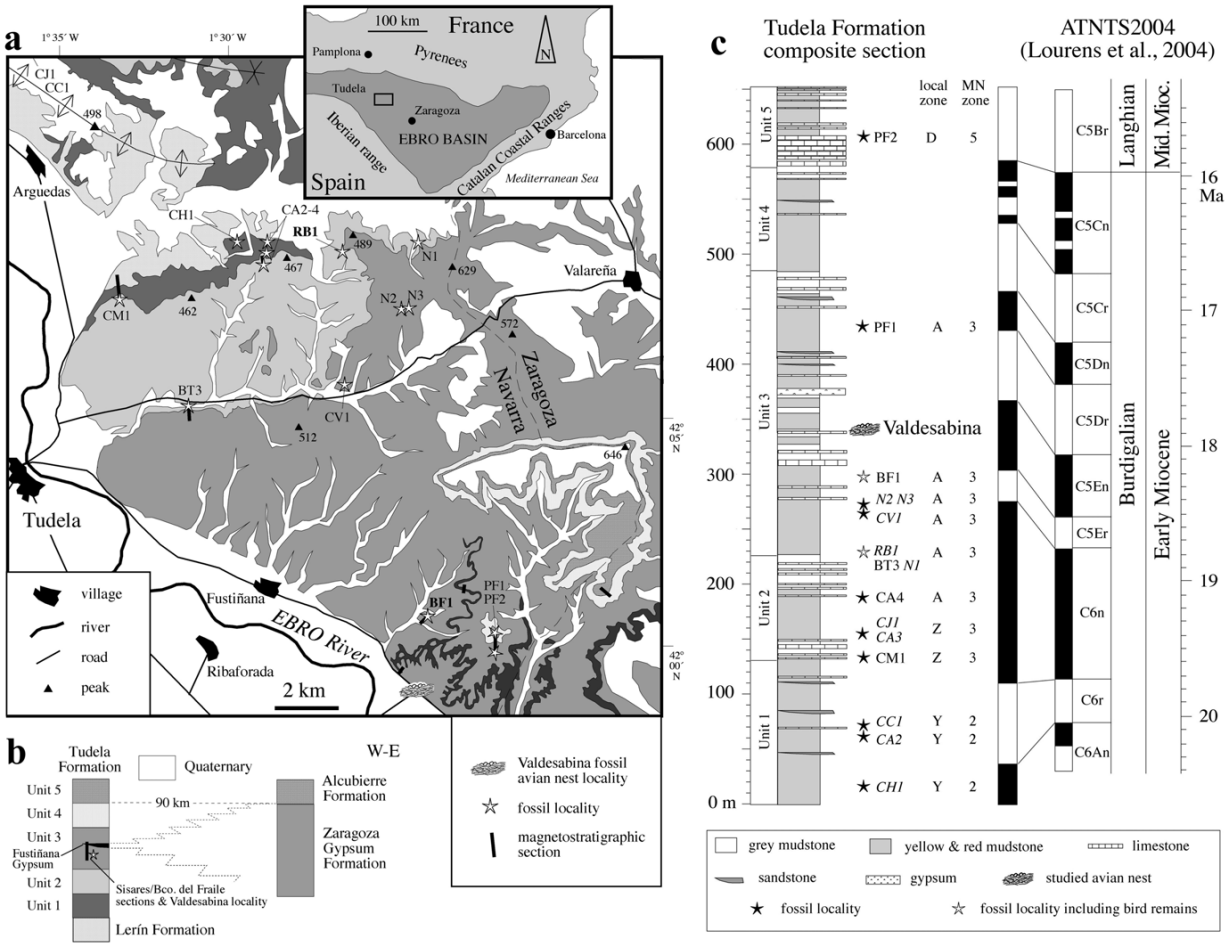
Site geology. (a) Location and geological sketch map of the Lower-Middle Miocene Tudela Formation in the Bardenas Reales de Navarra Natural Park, with location of the Valdesabina fossil avian nest locality studied in this work. (b) Scheme showing the lithostratigraphy of the Tudela Formation, with the position of the Vasldesabina fossil avian nest locality and the Sisares and Barranco del Fraile sections, and its lateral eastward gradation to the Zaragoza Gypsum Formation. (c) Composite magnetostratigraphic log of the Tudela Formation [3] and its correlation to the ATNTS2004 [14]. The location of fossil localities within the Tudela Formation is also shown (italics denote fossil localities correlated to, but not placed within, the studied magnetostratigraphic sections). All fossil localities from the Tudela Formation contain avian eggshell fragments.

The nest is in a limestone block (Vs-1) that comes from a flat-lying, 0.7 m-thick lacustrine carbonate level, which was quarried at the Valdesabina locality and belongs to Unit 3 [3] of the Tudela Formation (Figure 1). The Valdesabina limestone is a 70 cm-thick bed that is embedded in grey marls. Three and 1 thinner (<15 cm) additional limestone beds are interlaced within the marls just above and below, respectively, the main carbonate bed. Both the marls and the limestone beds are massive and contain remains of gastropods, ostracods and charophytes. The main limestone bed consists, in turn, of a lower 20 cm-thick stratum (named Bed 1) that is separated from the upper thicker (50 cm) stratum (named Bed 2) by a very faint contact layer. Bed 2 displays fairly abundant root marks that penetrate to 4–10 cm down from its top into the limestone. Grey marls beneath Bed 1 display horizontal lamination and include three thin (10–15 cm thick) intercalations of sandy marls. Reddish and brownish massive mudstones with frequent nodular gypsum nodules and some mottling are found below and above the grey marly interval, in which the Valdesabina limestone is located. This facies arrangement corresponds to facies association “B” of Arenas & Pardo [5], and indicates the development of a shallow lacustrine area with permanent water supply (marls and limestones) over a distal alluvial mudflat undergoing some evaporitic pumping (reddish and brownish mudstones). Subsequent filling and shallowing of the lacustrine area resulted in the establishment of palustrine conditions (root bioturbation) and, eventually, to the progradation of distal alluvial mudflats and deposition of reddish mudstones at the top of the sequence.

Lithostratigraphic correlation of the Valdesabina level to the neighboring (1 km apart) Sisares and Barranco del Fraile magnetostratigraphic sections [3] indicates that it has an estimated age of 18.3 Ma (middle part of chron C5En Burdigalian, Early Miocene [14]). Regarding the structural setting, the Valdesabina locality is characterized by the presence of several normal faults that visibly cut across the Valdesabina limestone level. These N-S trending faults are characterized by steep dipping fault planes (>70°), with small vertical offsets of less than 70 cm and sub-vertical striation pitches [4]. These faults often display undulated surfaces and are sometimes infilled with gypsum and calcite, indicating that they enabled the upward circulation of underlying formation waters (sulfate brines) when the limestone beds were only partially lithified. Most of these fluids corresponded to formation waters expelled during sediment compaction, but included also a significant fraction of waters advected laterally from marginal saline lakes that were, in turn, fed by hydrothermal activity at the front of the Iberian Ranges basal thrust [16].

### Nest

**Figure 2:**
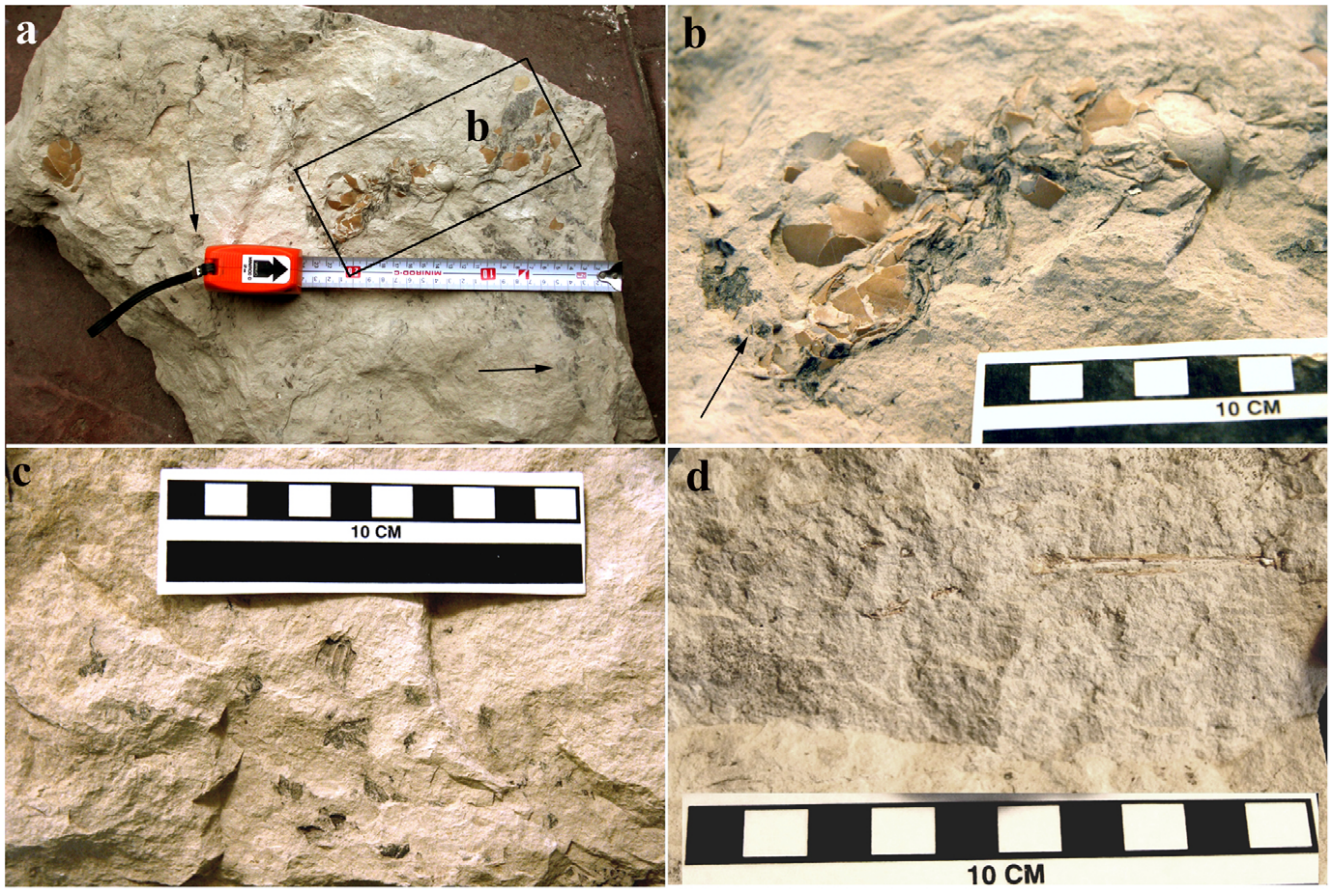
Nest and egg descriptions. (a) The limestone block Vs-1 with nest and eggs *in situ*. The exposed surface is interpreted as being at 10–15 cm from the upper side of Bed 2 of the Valdesabina Limestone. One egg is exposed in the upper left of the figure a few cm from the main concentration or clutch enclosed in a rectangle. The block is correctly orientated with the eggs on its upper surface. Arrows point to cf. Fabaceae material. (b) Detail of the main concentration of eggs. (c) Detail of the vegetable matter forming the nest material (d) A fragmentary hatchling tibiotarsus exposed in section in the side of Vs-1.

**Figure 3:**
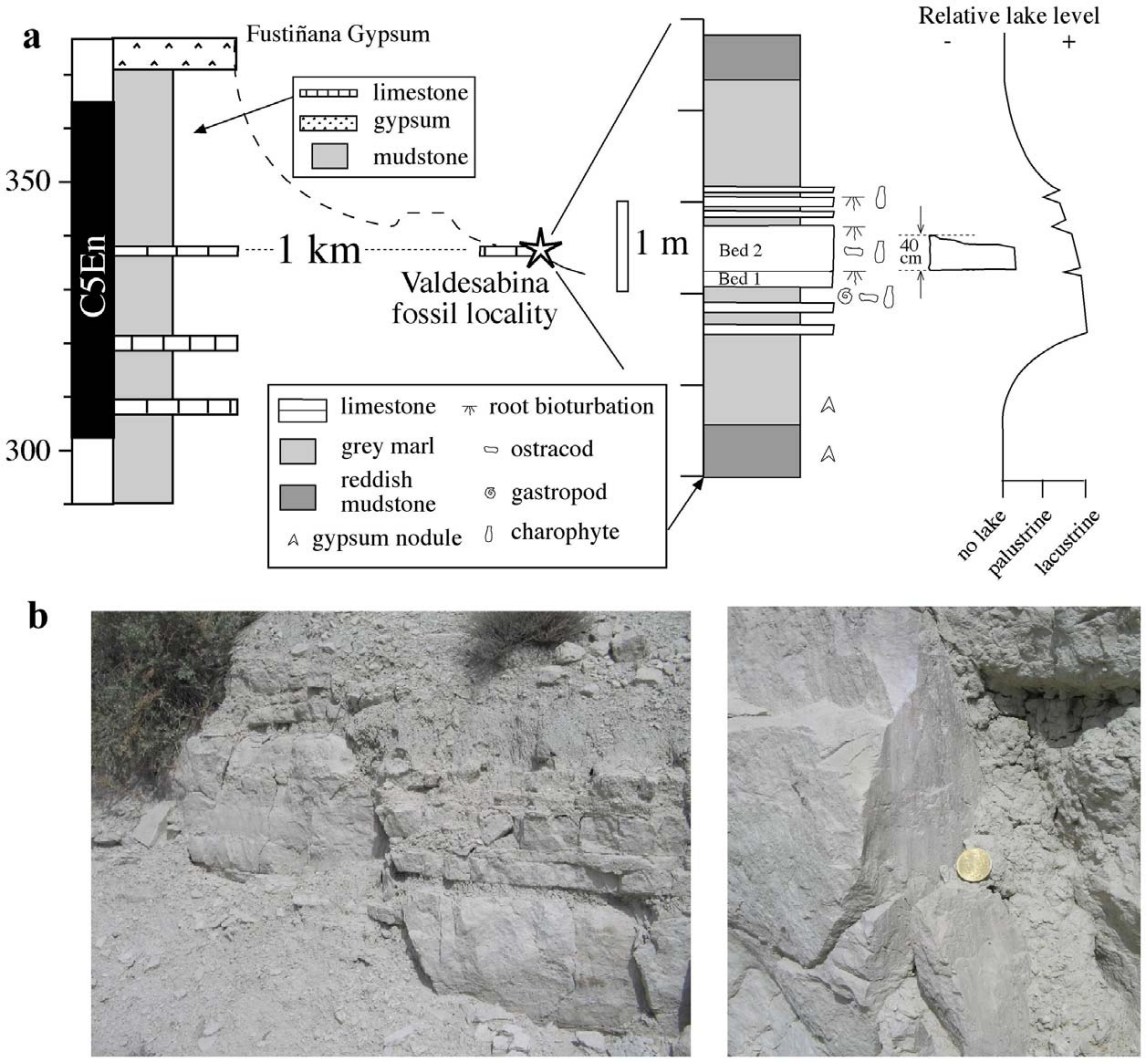
Geological context of the fossil nest. (a) Sedimentary log of the Valdesabina locality and its correlation to the neighbouring Sisares and Barranco del Fraile sections, for which magnetostratigraphic data are available. Reconstructed lake level variations are based on sedimentological and paleontological (ostracod) data (see also text). (b) Field examples of some of the normal faults that cut the Valdesabina limestone level (left) and a detailed view of the vertical striation pitches observed on the surface of one of these steeply dipping normal faults (right).

The Block, Vs-1, had an irregular shape with a maximum length of 55 cm, a width of 31 cm, and a maximum (before being cut for handling) thickness of 40 cm (Figure 2). The upper side of the block has an irregular surface (Figure 2a), which contrasts with the rather smooth surface of its opposite side. Given the thickness of the block, it is readily inferred that it derives from Bed 2, which is the only limestone bed in the Valdesabina locality with a thickness exceeding 20 cm (Figure 3a). In one of its sides, Vs-1 displays a steeply dipping fault with (sub)-vertical striation pitches and synthetic Riedel secondary microfractures. This fault enables determination of the sedimentary polarity of the block, thereby indicating that the smooth lower surface of the block corresponds to the base of Bed 2 and that the surface shown in Figure 2a is a subhorizontal plane through the upper levels of Bed 2 (Figure 3b). The clutch consists of at least five eggs (Figure 2a,b) exposed on the upper, irregular surface of the block. These eggs lie above a concave-up mat of preserved vegetal lining (Figure 2a,b) and concentrate in the upper 15 cm of the block and mostly cluster within a 10 cm radius in the centre of the block directly above the vegetal lining. The nest itself, as preserved, consists of carbonaceous twigs with distinctive leaves and leaflets. The vegetal material seems to have been slightly disaggregated. Judging by the present position of the twigs and leaves, the nest would not have exceeded a 600 cm^2^ area. The preservation of this fossil assemblage, its components, and its fragility prevented conventional mechanical preparation methods. Furthermore, CT-scan imaging has not yet enabled delineation of the exact dimensions and geometry of this fossil assemblage due to the lack of density contrast between the rock matrix and nest.

The taxonomic identification of the vegetal lining rests on the leaves that are twice pinnately compound leaves with (about) 1 cm long leaflets. These characters suggest an affiliation with the Fabaceae, a leguminous family that comprises more than 740 genera and 19,000 species (Figure 2c). This vegetal family [17] has leaves and stems very different from the structure of the algal (charophyte) communities characterizing the lake, thus suggesting a para-autochthonic origin for this building material. Therefore, the presence of this vegetation of a continental origin in a shallow lake characterized by a very low energetic sedimentary environment, combined with five (or more) incomplete eggs, strongly support the interpretation of a nest rather than an *ad hoc* assemblage of floating cf. Fabaceae material and eggs. Given the maximum thickness of Vs-1 (40 cm) and that of Bed 2 (50 cm), the eggs are emplaced very near (10–15 cm) to the bioturbated top of Bed 2. This indicates that the nest was laid in the latest stages of the evolution of the perennial lake but before its full filling and the concomitant establishment of palustrine (<50 cm water depth) conditions. One of the sides of the block was broken off 10–15 cm away from the nest (see following sections), exhibiting a few fragments of fossil avian bones (Figure 2d).

### Eggs

The five eggs exposed on the surface of Vs-1 consist of endocasts covered with a thin and exquisitely preserved eggshell layer (Figure 2a,b). One egg is exposed a few centimetres away from the main concentration or clutch (Figure 2). This observation, coupled with the minor disaggregation of the nesting vegetal material and sedimentological evidence for extremely low paleoenvironmental conditions, suggest that the nest sunk in the shallow lake without transportation. Observations of the endocasts and eggshell-covered endocasts indicate that the eggs were semi-elongated with a maximum length and width of 4.5 cm and 3 cm, respectively. The lack of embryonic remains *in ovo* coupled with the incomplete eggs suggest either that hatching had already occurred or that the nest was abandoned. No eggshell calcium resorbtion, usually observed in later embryonic ontogenetic stages, was noted during the eggshell microcharacterizations. Therefore, it would seem plausible that the nest was abandoned before sinking and being embedded in carbonate muds, as the palustrine area was filled during its subsequent evolution.

### Eggshell

**Figure 4:**
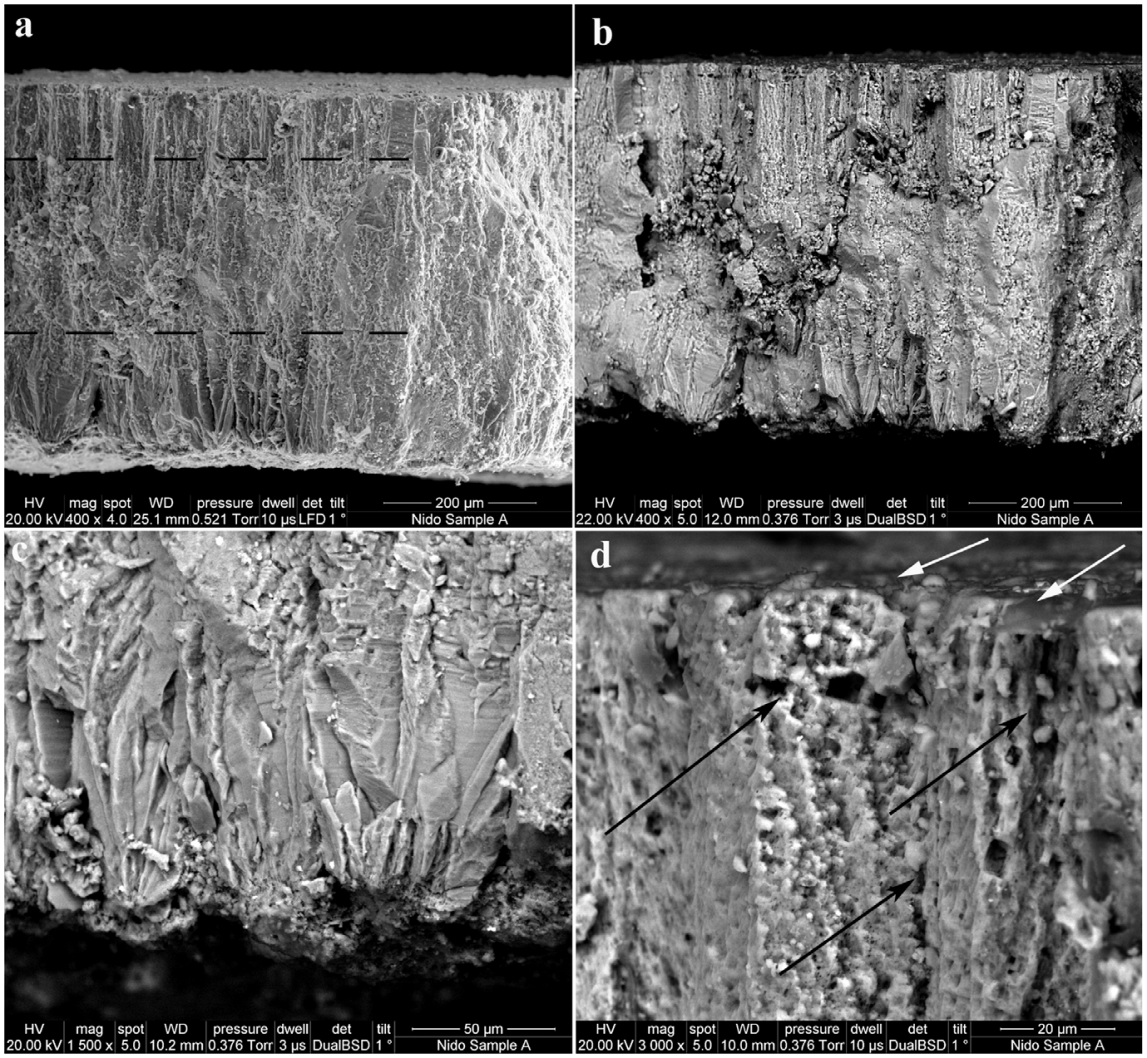
SEM microcharacterizations. (a) SEM radial sections reveal a three laminated eggshell structure, indicative of an avian eggshell, with an average total thickness of 470 µm, consistent with Kohring [20] observations on modern flamingos. The 163 µm thick layer 1 consists of elongated calcite crystals that radiates mostly outward from nuclei at the eggshell base. The 262 µm thick layer 2 is characterized by a crystallographic arrangement only easily described with EBSD microcharacterization (Figures 5 a and b). The 63 µm thick layer 3 displays long rectangular crystals with a spongy appearance due to multiple voids in the crystalline structure. (b) Backscattered electron microscopy (BSEM) of the same eggshell fragment. BSEM allows a better observation of the outer most eggshell surface covered with a thin calcium phosphate granular layer that does not appear to be a diagenetic artifact. Note the well-defined basal crystals and obvious large pore canals, which by themselves indicate a high moisture level in the nest, here congruent with the floating nest. (c) SEM of the basal crystals shows clearly well-defined spherulitic crystals that surround the nuclei. (d) BSEM of the same specimen upper eggshell section reveals the spongy appearance of this eggshell, the high concentration of micro size pore canals (black arrows), most of which seem to abut below the outer-most thin covering to indirectly connect with the eggshell surface. The sum of these microscopic features support a flamingo type eggshell and egg. The white arrows point to the granular relief of the surficial layer.

**Figure 5:**
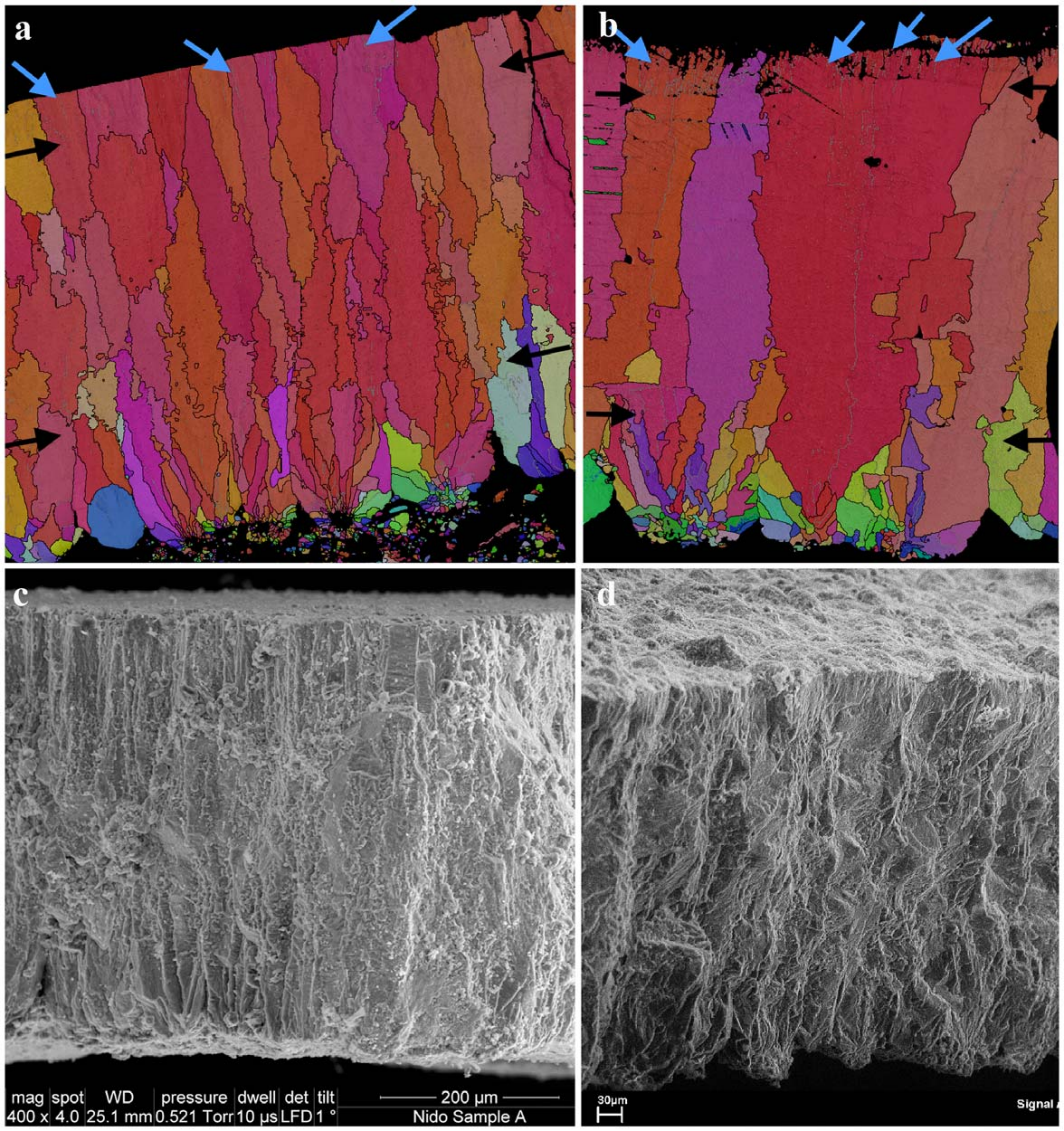
EBSD microcharacterization. (a) An EBSD orientation map of a cross section through the Vs-1 eggshell, with the outer surface of the eggshell at the top of the image and non-indexed regions marked in black. The colour scheme relates to the orientation of the calcite crystal lattice, with red colours indicating an alignment of the c-axis with the eggshell surface normal. High angle grain boundaries (>10° misorientation) are marked by black lines and low angle boundaries (2–10° misorientation) by grey lines. The grain structure can be subdivided into 3 distinct layers, with layer boundary positions highlighted by the black arrows. Numerous low angle boundaries are present in the outermost layer, some of which are marked by blue arrows. (b) A comparable EBSD orientation map of a cross section through *P. ruber* eggshell, using the same colour scheme as in (a). This shows many of the same features visible in (a), including the presence of 3 distinct layers (marked by black arrows) and an abundance of low angle boundaries in the outermost layer (blue arrows). The boundaries between EBSD grain structure seem to mirror the eggshell structural layer boundaries. Some subtle differences in the crystallographic structure between these extinct and modern eggshell are visible such as the less serrated nature of the grain boundaries in the middle layer and the greater spacing of the individual shell units in the modern specimen. (c) and (d) SEM photomicrographs of fractured cross sections through through the Vs-1 and *P. ruber* eggshells respectively. These images complement the EBSD orientation maps, highlighting the 3-layered structure of both eggshells but lacking the detailed grain structure information visible in (a) and (b). The scale bars in a, b, and c images are 200 µm.

The eggshell micro-characterization was performed with Scanning Electron Microscopy (SEM) and Electron Back Scatter Diffraction (EBSD), two complementing techniques that reveal a wealth of microscopic features paramount for taxonomic identification [18] and functional morphology [19]. Several eggshell samples were collected from the Vs-1 eggs and also from other fossil localities throughout the Bardenas Reales (Figure 1c). Microscopic examinations of the eggshell specimens from the Tudela Formation indicate that the preserved fossil avifauna in this basin was monotypic, which is further confirmed by a monospecific assemblage of avian bones (see following section). As such, the specimen from the nest will be used for descriptive purposes, as they are the best-preserved samples due to the lack of transport. SEM radial sections reveal a three laminated eggshell structure (Figures 4 and 5) with an average thickness of 470 µm, a measurement consistent with Kohring's [20] observations on modern flamingo eggshells. The 163 µm thick layer 1 consists of elongated calcite crystals that radiate outwards from nuclei at the eggshell base (Figure 4c). Overall, these basal crystals give a long and slender aspect to the eggshell units (Figure 4a,c). The 262 µm thick layer 2 is characterized by a crystallographic arrangement not easily described using SEM microcharacterization (Figure 4a,b). The 63 µm thick layer 3 displays long rectangular crystals with a spongy appearance due to multiple voids in the crystalline structure (Figure 4d). The outermost eggshell surface is covered with a thin granular layer that does not appear to be a diagenetic artifact (Figure 4d). This eggshell type is further characterized by a high pore concentration that either reaches its outer surface or abuts against the outermost granular layer (File S1). The eggshell macro and micro-structures closely match in several aspects that of the modern flamingo *Phoenicopterus ruber* (Figure 5), the common American Flamingo, and is congruent with previous SEM observations [20]. To further test the affinities of the eggshell structures and microstructures, Vs-1 was compared within selected bird eggshells, namely with those of putatively closely related taxa [21], [22]. In addition to *P. ruber*, eggshell observations included the Western Grebe (*Aechmophorus occidentalis*), White Ibis (*Eudocimus albus*), Spoonbill (*Platalea ajaja*), White Stork (*Ciconia ciconia*), and a Great Bustard (*Otis tarda*). SEM analyses in conjunction with EBSD microcharacterizations revealed that Vs-1 eggshell characters and character-states are most shared with *P. ruber* in contrast to the other birds (File S2). Considering the recently supported grebe-flamingo sister taxon relationship [22], [23], we further compared the eggshell dimensions and microstructures of samples from Vs-1 with those of grebes, and flamingos. The total eggshell thickness of Vs-1 (470 µm) closely matches that of *P. ruber* (466 µm) but greatly differs from that of *A. occidentalis* (258 µm). The Vs-1 shell microstructure is trilaminated and covered by a thin outer granulose layer, characters present in the both modern taxa. Although layer 1 spherulites and layer 2 columns are quite similar in Vs-1 and *P. ruber*, they exhibit minor variations that are autopomorphic to each taxon and quite visible by EBSD analyses (see below). Overall, the layer 2 columns are seemingly wider in extant flamingo, and their spherulites are not as pronounced as in Vs-1 shell. Layer 3 in the shells of Vs-1 and *P. ruber* is quite similar in morphology and size (63 µm). However, these features differ tremendously compared to *A. occidentalis*, in which the layer 3 is only 30 µm thick, the layer 2 consists of wide cylindrical-like columns and the layer 1 spherulites are extremely bulky. Orientation maps generated from the EBSD data are shown in Figure 5. There is little indication in the EBSD results for significant diagenesis in the fossilized sample (File S1). Only the circular, blue coloured grain in the lower left of the map (Figure 5a) shows the characteristic features of diagenetic alteration, namely a lack of low angle boundaries, a different grain morphology from the surrounding structure and a better quality of diffraction pattern. The maps from the two samples (Vs-1 and *P. ruber*) display many similarities (Figs. 5a, 5b). In both cases, the shell cross section can be divided into 3 distinct layers, as in the SEM images. The innermost layer shows distinct nucleation points spaced approximately every 50–100 µm, with calcite grains radiating outwards in a fan-like structure. This structure continues for approximately 100 µm in both samples, before a gradual transition to a second layer. The layer 2 is characterized by elongate grains extending outwards towards the external surface of the shell, with a dominant calcite crystal orientation of the c-axis aligned perpendicular to the outer surface. The boundaries between the grains in layer 2 are very irregular (black lines in Figure 5a), with many small lateral offsets. The outermost layer 3 is characterized by a significant increase in the number of low angle boundaries (grey lines Figure 5a), oriented perpendicular to the outer shell surface. The boundary between layer 2 and layer 3 is relatively indistinct (Figure 5a) but sharper in the comparative modern flamingo shell (Figure 5b). Layer 3 is about 50–60 µm thick in both samples and can also be clearly seen in the secondary electron SEM images of the fractured shell cross sections (Figure 5c,d). However, there are some notable differences between the eggshells of Vs-1 and *P. ruber* that are highlighted by the EBSD results. The radiating fan-like structure in layer 1 is more clearly pronounced in Vs-1 than in *P. ruber* shell; the grain size of the modern specimen is significantly larger and its density of low angle boundaries in layer 3 is higher than Vs-1 shell. Overall, the EBSD results are congruent with SEM observations and suggest that these two eggshells share enough derived characters to belong to the same clade but are not the same species. The most notable difference is that the width of the crystals in layer 2 seems wider in *P. ruber* than Vs-1 shell.

### Osteology

**Figure 6:**
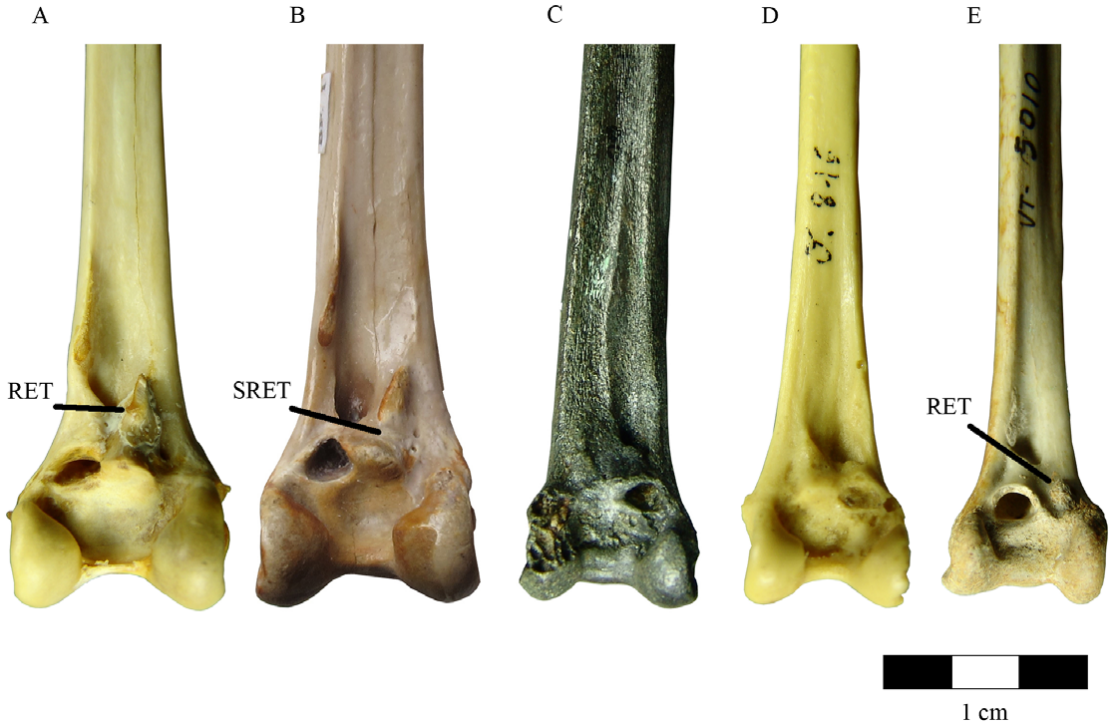
Comparison of the Palaelodidae and Phoenicopteridae distal tibiotarsi. (a) *Phoenicopterus ruber* (MZUSP 88485); (b) BAS new species; (c) *Agnopterus sicki* (MHNT 4257); (d) *Phoenicopterus croizeti* (MHNT 5085); (e) *Palaelodus* spp. (MHNT 5010). The most diagnostic characters found in BAS are a crest in the *Retinaculum Extensorium Tibiotarsi* (RET) divided by a deep and conspicuous sulcus from the articular facet for the intercotylar eminence of the tarsometatarsus (b), a deep intercondylar incision extending over the lateral condyle, a tuberosity for the RET medial attachment and a deep sulcus surrounded by crests cranial to the *trochlea cartilaginis tibialis* (not shown). RET: *Retinaculum Extensorium Tibiotarsi*. SRET: sulcus between the RET and the articular facet for the intercotylar eminence of the tarsometatarsus in the BAS specimen.

Monospecific fossil bird remains have been recovered from two sites in the Tudela Formation (Figure 1), Rincón del Bú 1 (RB1) and Barranco del Fraile 1 (BF1) indicating a recurrent presence of this avian lineage. The most significant bone fragment found with Vs-1 is a hatchling tibiotarsus that was post-burially sectioned longitudinally (Figure 2d). Its taxonomic identification is dubious due to its early ontogenetic developmental stage and preservation. Yet, its overall morphology and cnemial crest shape suggest a possible Phoenicopteridae taxonomic affiliation. The osteological material was first tentatively attributed to Phoenicopteridae [9]. A distal left tibiotarsus (BF1-1) (Figure 6b) and a distal left tarsometatarsus (BF1-3) lacking *trochlea metatarsi* II provide sufficient diagnostic characters to confidently place these two Bardenas specimens (hereafter refer as BAS) within Phoenicopteridae Bonaparte, 1838 [24]. Several genera and species of Phoenicopteriformes have been defined based on the features of tibiotarsus [21], [25], [26]. The assignement of BF1 and RB1 specimens to Phoenicopteridae rests on the presence of several features in BF1-1 on the distal rim of its *condylus lateralis* and *medialis*: in cranial view, the distal portion of tibiotarsus presents a wider and more medially located *sulcus extensorius*, a longer supratendinal bridge (in comparison with Palaelodidae), a distinct projection caudally of the lateral and medial condyles from the shaft; a distinct and conspicuous sulcus proximal to the medial condyle that separates it from the distal opening of the supratendinal canal and a lack of a medial ridge in the *trochlea cartilaginis tibialis* (see [22], [27]). Representatives of Palaelodidae (*sensu* [27]) are diagnosed by a rounded and prominent tuberosity for the lateral attachment of the *Retinaculum Extensorium Tibiotarsi* (hereafter RET) (Figure 6e), while in Phoenicopteridae the RET is a conspicuous crest. BAS differ from the other phoenicopterids studied by having this crest divided by a conspicuous and deep sulcus from the articulation facet for the intercotylar eminence of the tarsometatarsus (Figure 6b) and by having the lateral attachment of RET laterally to the *sulcus extensorius* rather than alongside and partly overlapping the *pons supratendineus*. *Phoenicopterus* and *Phoeniconaias* present essentially the same structures in the tibiotarsus, only differing by their relative sizes. Like the crown groups *Phoenicopterus* and *Phoeniconaias*, BAS possesses a wide and medially located *sulcus extensorius*, in contrast to Palaelodidae, the closest sister taxon to Phoenicopteridae, where it is narrow and centrally located in the shaft. Moreover, BAS displays a deep intercondylar incision that extends over the medial condyle, similar to *Phoenicopterus* and *Phoeniconaias* (Figure 6a,b). This intercondylar incision also markedly excavates the proximomedial side of the lateral condyle as it does in phoenicopterids, in contrast to palaelodids but extending in a lateral condyle to the diaphysis. Other Phoenicopteridae have a ridge for the RET medial attachment in the medio-distal portion of *sulcus extensorius*, replaced whereas in BAS by this attachment is a tuberosity for the RET medial attachment. In contrast, Palaelodidae are characterized by a shallow sulcus cranial to the *trochlea cartilaginis tibialis*. Yet, BAS has a deep sulcus surrounded by crests. In BF1-3, the *trochlea metatarsi* III, in plantar view, displays a conspicuous crest, not found in the Palaelodidae and other Phoenicopteridae. Given the present data, BAS is best interpreted as a taxon basal to the Phoenicopteridae crown clades (*Phoenicopterus*, *Phoeniconaias* and *Phoenicoparrus*), rather than a palaelodid. Regarding its size, it is worth noting that BAS measurements are a mosaic of those from *Phoenicopterus ruber* and *Phoenicopterus chilensis* (Table 1).

**Table 1:** Measurements (mean and standard deviation) of selected bone features in fossil and extant Phoenicopteriformes.

Taxa	Width of distal epiphysis	Height of lateral condyle	Height of medial condyle	Width of distal diaphysis	Heigth of distal diaphysis	Intercondylar distance	Width of Trochela III
*Phoenicopterus ruber*	17,09 (1,80)	19,20 (1,83)	18,86 (1,50)	09,73 (0,55)	07,76 (0,66)	05,14 (0,22)	07,73 (0,80)
BAS	17.8	16.4	16	8	6.5	4	7
*Phoenicopterus chilensis*	15,56 (1,11)	16,71 (1,08)	16,71 (1,04)	09,05 (0,66)	06,65 (0,52)	04,84 (0,45)	07,09 (0,50)
*Agnopterus sicki*	14	16.9	15.5	9.5	6.7	4	-
*Phoenicopterus croizeti*	13.09	16.9	15.9	8.6	6.3	3.8	6.5
*Phoeniconaias minor*	12,92 (0,85)	14,46 (0,96)	14,46 (0,80)	07,71 (0,38)	05,81 (0,28)	04,02 (0,50)	05,93 (0,51)
*Palaelodus* spp.	12,61 (0.68)	13,48 (1,09)	07,04 (0,98)	07,04 (0,49)	04,97 (0,45)	03,96 (0,70)	05,30 (0,21)

BAS measurements suggest the size of new species would have been intermediate to Phoenicopterus chilensis and P. ruber. Number of specimens analyzed after the scientific names and all values given in millimetres.

### Paleoenvironmental reconstruction of the nesting site

A total of 78 ostracod shells and shell fragments have been extracted from the laminated lacustrine marls located just underneath the Valdesabina limestone level, of which 47 could be classified (See Methods). The association of ostracods, dominated by *Ilyocypris gibba* (21 shells, 44.7%) and *Paralynocythere* sp. (13 shells, 27.7%), with smaller numbers of *Candona* cf. *spelaea* (7 shells, 14.9%) and *Limnocyithere* sp. (6 shells, 12.7%), points to a permanent, shallow (0.5–6 m) endorheic lake characterized by oligohaline (seasonally mesohaline) and suboxic conditions developed under a prevailing warm and arid climate. Such climatic context is fully congruent with the presence of ectothermic faunas (e.g. crocodilians, turtles, amphisbaenians and erycine snakes) in fossil localities [9], [10] throughout the Tudela Formation. Ostracod fauna suggest a water depth of 0.5–6 m for the marls underlying the Valdesabina limestone level, which represents the deepest member of the shallowing-upwards sequence. The stratigraphic position of the nest in the upper part of the overlying Valdesabina limestone bed indicates that the nest was laid in the latest stages of the filling of this perennial shallow lake (Figure 3a), hence in a shallower water depth, but before the full establishment of palustrine conditions (e.g. 0.1–0.2 m). Overall, this points to a water depth of less than 1 meter.

Organic geochemistry data (See Methods) indicate that both the nest plant material and the limestone are characterized by light *n*-alkanes, which is consistent with the occurrence of both spores and algal cell walls [28]. The predominance of even carbon numbers, along with pristine/phytane ratios of <1 in the studied material, suggest suboxic conditions were present during accumulation and depletion of organic matter in the nest, probably in concurrence with saline conditions [29]. This indicates that suboxic and oligohaline (perhaps seasonally mesohaline) conditions prevailing during the life of the former perennial lake, as inferred from ostracod faunas, continued in the transition to palustrine conditions in which the nest was made. The additional occurrence of homologous heavy *n*-alkanes with a predominance of odd carbon numbers in the nest material indicates a link with long-chain n-alkanes derived from near shore plants [29], which is entirely consistent with the provisional taxonomic identification of cf. Fabaceae. ^87^Sr/^86^Sr composition of eggshell fragments from the nest (^87^Sr/^86^Sr = 0.708693 (±6 SE, standard error in ppm), ^87^Sr/^86^Sr = 0.708703 (±7), and ^87^Sr/^86^Sr = 0.708712 (±7) and that of the embedding limestone (^87^Sr/^86^Sr = 0.708704 (±6) and ^87^Sr/^86^Sr = 0.708696 (±5) (See Methods) are statistically identical. This indicates that the eggshell fragments were in equilibrium with the lacustrine carbonate. In view of the lack of a significant diagenetic overprint on the eggshell fragments from the nest, the most likely explanation is that both the eggs and the parent lacustrine limestone were linked to the same Sr source constituted by the paleolake water. This indicates that the BAS feeding and nesting behavior was intimately linked to the lacustrine area, with no detectable influence of perilacustrine areas.

## Discussion

### Phylogenetic, biological, and ecological implications for the Phoenicopteridae evolution

**Figure 7:**
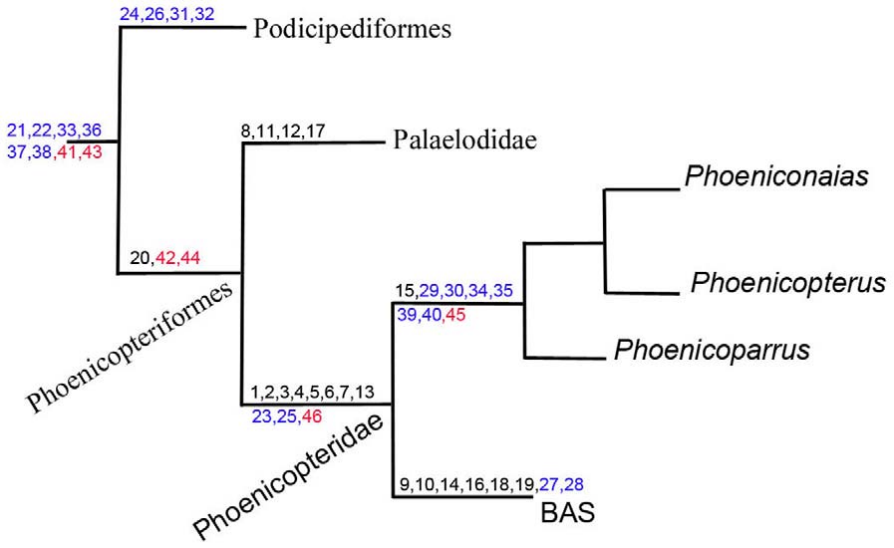
Osteological, oological, nesting strategy, and ecological characters mapped onto a phylogenetic tree modified from Mayr [**23**] by the insertion of the BAS specimen to show hypothesised character transitions on the flamingo lineage. Characters come directly from the text. Black, blue, and red numbers illustrate osteological, oological/nest, and environmental characters, respectively. OSTEOLOGICAL CHARACTERS (black numbers): Tibiotarsus: 1: distinct notches on the distal rim of *condylus lateralis* and *medialis*; 2: a wide *sulcus extensorius*; 3: a medially located *sulcus extensorius*; 4: a longer supratendinal bridge (in comparison with Palaelodidae); 5: a distinct projection of the lateral and medial condyles from the shaft; 6: a distinct and conspicuous sulcus proximal to the medial condyle; 7: lack of a medial ridge in the *trochlea cartilaginis tibialis*; 8: a rounded and prominent tuberosity for the lateral attachment of the *Retinaculum Extensorium Tibiotarsi* (RET). *Retinaculum Extensorium Tibiotarsi* separated from the articular facet of the intercotylar eminence by: 9: conspicuous sulcus; 10: deep sulcus; 11: narrow *sulcus extensorius*; 12: lateral attachment of RET laterally to the *sulcus extensorius*; 13: deep intercondylar incision; 14: intercondylar incision extends over lateral condyle to the diaphysis; 15: ridge in the medio-distal portion of *sulcus extensorius*; 16: tuberosity for the RET medial attachment; 17: shallow sulcus cranial to the *trochlea cartilaginis tibialis*; 18: deep sulcus surrounded by crests. Tarsometatarsus: 19: a conspicuous crest in *trochlea metatarsi* III, in plantar view. Skull: 20: presence of salt glands. EGGS AND NESTS (blue numbers): 21: trilaminated eggshell structure; 22: thin outer granulose calcium phosphate layer; 23: eggshell thickness of or approximately 466 µm; 24: eggshell thickness of or approximately 258 µm; 25: Layer 3 of or approximately 63 µm; 26: Layer 3 of or approximately 30 µm; 27: thin columns in L2; 28: Pronounced spherulitic crystals in L1; 29: Wide columns in L2; 30: moderately pronounced spherulitic crystals in L1; 31: Large cylindrical columns in L2; 32: Bulky spherulitic crystals in L1; 33: 45×30 mm eggs; 34: 90×53 mm eggs; 35: Single egg per clutch; 36: Two to seven eggs per clutch; 37: Floating nest; 38: Twig-nest; 39: Ground nest; 40: Mud, volcano-shaped nest. NESTING ENVIRONMENTS (red numbers): 41: Nesting in lacustrine environment; 42: Nesting in endorheic lacustrine environment; 43: Nesting in normal-oligohaline lacustrine environment; 44: Nesting in oligohaline to mesohaline lacustrine environment; 45: Nesting in mesohaline to hyperhaline lacustrine environment; 46: Nesting in shallow to extremely shallow lacustrine environment.

Van Tuinen *et al.* [30] proposed a sister group relationship between flamingos (Phoenicopteridae) and grebes (Podicipedidae) based on molecular analyses. Mayr [22] further substantiated this relationship with a phylogenetic analysis including 70 morphological characters. Although this sister group relationship is now well-supported, flamingos and grebes exhibit feeding, reproductive, and nesting strategies that diverge significantly. Flamingos are long-legged filter feeders, grebes are diving birds [31]. Flamingo nesting behaviours are characterized by ovideposition of a single egg (∼90×54 mm) in a muddy volcano-like nest, whereas grebes typically build floating platform nest, anchored to the lake bottom with aquatic plants, and with 3 to 8 (∼43×30 mm) eggs [32]. Their respective eggshell morphologies are substantially different and could be easily identified under SEM examinations [20]. The earliest, well-preserved fossils of grebes are from Miocene deposits [33], [34], [35], [36]. Flamingo and their allies have an extensive fossil record dating from the Oligocene [37], [38]. Conversely, Palaelodidae are known from numerous skeletal remains from the Tertiary of Europe, Americas, New Zealand, and Australia [21], [27], [39], [40], [41] and were posited as a possible evolutionary link between Phoenicopteridae and Podicipedidae [22], [23]. Although palaelodids may bridge the morphological gap between extant grebes and flamingos [23], they do not provide any data about the extreme divergence in the reproductive and nesting behaviours nor the ecologies between these two avian sister clades. The fossil bones (BAS) display osteological characters that suggest this new species was a phoenicopterid, sister taxon to all the extant Flamingo genera (Figure 7). Egg shapes [42], [43], [44], eggshell microstructures [43], [44], and eggshell DNA [45] are all genetically programmed and display characters that are species-specific [46]. Vs-1 eggshell microcharacterizations support a phoenicopterid taxonomic identity. However, the nest structure, the number of eggs in this clutch, and the egg shape and size compare favourably with grebes' reproductive characters. The Vs-1 nest was built from cf. Fabaceae twigs and leaves, rather than the expected reeds or cattail palustrine flora. The Vs-1 nest floated in an extremely shallow endorheic lake, only a few centimetres from the bottom. As such, the nesting behaviour inferred from the Vs-1 fossil represents an interesting evolutionary step intermediate to the Podicipedidae and Phoenicopteridae crown groups indicating that a floating nest is a plesiomorphic condition for Miranornithes (Podicipediformes+Phoenicopteriformes [47]), which transformed from the Vs-1 condition in basal Phoenicopteridae (and likely Palaelodidae) to a derived volcano-nest build built from lake sediments on lake substrates in the modern flamingos [31]. Extant flamingos are known to lay a single large egg (File S3). However, grebes commonly lay two to seven egg in a clutch [32], with the eggs having similar dimensions (45×30 mm) to those of the eggs in Vs-1 (File S3). Considering Vs-1/BAS condition, the ovideposition of a single and larger egg should be considered autapomorphic for the modern flamingos, while smaller egg sizes and higher egg numbers per clutch should be regarded as plesiomorphies for Miranornithes. Interestingly, the eggshell structures differ extremely between extent grebes and flamingos in their internal crystallographic arrangement, although the thin granulose calcium phosphate outer surficial layer [23] seems shared by both lineages [20]. Vs-1 eggshell displays clear affinities with flamingo eggshell (see the descriptive section) and markedly differs from grebe eggshell structures [20]. As such, this new fossil indicates that the flamingo-type eggshell structure is synapomorphic for BAS and other Phoenicopteridae crown clades but is not shared with Podicipediformes. Nests of modern flamingo species are commonly built in very shallow lacustrine saturated to hypersaturated environments in respect to salts and alkaline minerals [31], [48]. The inferred palaeoecological conditions under which Vs-1 was deposited indicate that the nest was built in an endorheic lake with a suboxic environment saturated with respect to gypsum and calcite. Such an environment would have inhibited the growth of cattail and reed flora that normally represent the prime nesting material for modern grebes and their allies. Interestingly, these lacustine environments, coupled with oligosaline and suboxic conditions (and more exaggerated conditions), are preferred nesting grounds for modern flamingos (File S3) but, if less severe, could also sustain grebe feeding and nesting [48]. Although, flamingo and grebe nesting sites are not commonly shared in the northern hemisphere, these two lineages co-exist in similar ecological systems in the Andean punas [48]. Yet, despite inhabiting the same punas, their respective nesting environments are directly correlated with salinity gradients [48], with flamingos preferring hypersaline and grebes oligohaline conditions [31], [48]. Vs-1 geological, sedimentological, and paleoecological data indicate rhythmic accumulations of distal alluvial sediments in an endorheic lake driven by orbital forcing via changes in runoff. Moreover, geological evidence suggests that the gypsum-saturated waters that were expelled from the underlying sediments and forced through the existing syn-sedimentary faulting system, favoured above-normal gypsum concentrations in the lake system at the time of nesting. This might have ensured survival of microbialites and ostracods, which in turn ensured food and created favorable nesting ground conditions for BAS during the Miocene climatic optimum [49], a period recognized for abundance of neotropical avifauna in Europe [15]. In summary, the palaeoecological context of Vs-1 is congruent with the punas type environments in which both modern grebes and flamingos could thrive and reproduce. However, oligohaline (seasonally mesohaline) conditions prevailed in Bardenas and did not reach the hyper salinity of modern Phoenicopteridae nesting grounds. This conclusion is phylogenetically congruent with previous arguments [23] that palaelodids possessed a primitive filter feeder apparatus and salt glands. This indicates a switch from fish predation in grebes to the filter feeder behaviours in stem-phoenicopterids living in oligo or mesohaline environments, which were further developed in Phoenicopteridae crown clades (Figure 7).

The discovery of Vs-1 represents the first occurrence in the fossil record of a floating avian twig-nest, which is remarkable considering the building material and the fragility of such a structure. Even more remarkable, the nest contained at least five eggs *in situ*. The sum of geological and sedimentary evidence coupled with geochemical, organic and isotopic analyses strongly support the inference that the fossil represents a nest rather than an *ad hoc* assemblage of floating cf. Fabaceae material with eggs in a lacustrine environment. Furthermore, the eggshell and other observations made from Vs-1 provide new and unexpected phylogenetic data and shed light on the evolution, ecology, nesting strategies and habitats of Miranornithes, not available from previous studies. As such, it bridges the morphological, ecological, and oological gap between extant grebes, palaelodids, and extant flamingos. Our cross-disciplinary study that involved oological, osteological, and observations in conjunction with detailed geochemical, isotopic, and organic geochemistry analyses reveal that paleoflamingos shared several reproductive, nesting, feeding, and ecological behaviours with grebes and acquired their unique and derived characters progressively in their phylogeny. Moreover, our results indicate that favourable nesting environments consist of shallow lakes, as previously mentioned, but also with a minimum amount of saturation in respect with alkaline minerals and salts below which successful nesting habitats would become either problematic or unfeasible.

## Materials and Methods

### Bardenas avian osteological and oological fossils

Nest and associated eggs: Vs-1 curated provisionally at Departamento de Estratigrafía y Paleontología de la Universidad del País Vasco. Paleoflamingo fossil skeletons: Rincón del Bú 1 (RB1) curated provisionally at Departamento de Estratigrafía y Paleontología de la Universidad del País Vasco. Barranco del Fraile 1 (BF1) curated provisionally at Departamento de Estratigrafía y Paleontología de la Universidad del País Vasco. BF1-1: distal left tibiotarsus; BF1-3: incomplete distal left tarsometatarsus; RB1-196: incomplete right distal tibiotarsus; BF1-5: incomplete left cranial coracoideum; RB1-2: phalanx digitis majoris; RB1-188; fragment of rib; BF1-2: proximal radius; BF1-4 and BF1-6 vertebrae; BF1-7 head of humerus; BF1-13: podal phalanx.

#### Avian comparative osteolgy

The osteological terminology follows Baumel *et al.* [50].

Comparisons with fossil and recent skeletons are based in specimens housed both in Museu de Zoologia da Universidade de São Paulo (MZUSP) and Museu de História Natural de Taubaté (MHNT), Brazil. *Phoenicopterus ruber* (MZUSP 88485; MHNT 039; MHNT 10034); *Phoenicopterus chilensis* (MZUSP 88482; MZUSP 88486; MHNT 10048; MHNT 10051); *Phoeniconaias minor* (MZUSP 88483; MZUSP 88484; MHNT 1336); *Phoenicopterus croizeti* (MHNT 5085); *Agnopterus sicki* (MHNT 4257); *Palaelodus* spp. (MHNT 5005; MHNT 5006; MHNT 5007; MHNT 5008; MHNT 5010; MHNT 5015; MHNT 5016; MHNT 5018; MHNT 5019); *Podilymbus podiceps* (MZUSP 87909); *Balearica pavonina* (MZUSP accession number pending). Oological specimens housed at Museu de Zoologia da Universidade de São Paulo and The Field Museum of Natural History (FMNH). *Phoenicopterus ruber* (MZUSP 514); *Podiceps auritus* (MZUSP 621); *Podilymbus podiceps* (MZUSP 382–1; MZUSP 382-2:); *Podiceps major* (MZUSP 352); *Eudocimus albus* (MZUSP 185); *Platalea ajaja* (MZUSP 632)*; Otis tarda* (MZUSP 446); *Ciconia ciconia* (MZUSP 616); *Aechmophorus occidentalis* (FMNH 15452).

### Field data

Vs-1was fortuitously discovered while the limestone level was being quarried in order to construct a retaining wall for the February 2003 floods of the neighbouring Ebro River. In addition to the eggshells in Vs-1, we have also studied eggshell fragments recovered from the concentrated residue that resulted from washing and sieving of about 10000 kg of sediment collected from the 16 fossil localities found up to date in the Tudela Formation in search for vertebrate remains. Eggshell fragments are well preserved because no acid was used to aid in the extraction of fossil remains.

### EBSD Techniques

Microstructural analyses of the eggshell structures were carried out using electron backscatter diffraction (EBSD) on a Zeiss Ultra Plus FEG-SEM at the University of Sydney. EBSD is an SEM-based diffraction technique that measures the complete crystallographic orientation of the crystal lattice from a submicron area on the sample surface (e.g. [51], [52]) and that has only recently been applied to the characterisation of extinct dinosaur eggshells [18]. Scanning the electron beam across an orthogonal grid and measuring the crystal orientations at each point on the surface allows the grain structure of the sample to be reconstructed in the form of an orientation map. The microstructure is fully characterised using this automated approach, and therefore EBSD is widely applied in both the materials sciences and in geology (e.g. [53]). For this study, small fragments (approximately 5×5 mm) of each sample were mounted in epoxy; a cross section through the shell thickness was polished using diamond paste with a final stage using colloidal silica. The surface was then coated with a very thin gold coat (2–4 nm) and the sample was subsequently tilted at a high angle (70°) from horizontal for EBSD analyses. EBSD measurements were collected with a spacing of 0.5 mm on a grid of 1117×1128 points (Vs-1) and 1092×930 points (*P. ruber*), using an Oxford Instruments CHANNEL5 EBSD system. The data was collected in approximately 12–16 hours, at a rate of between 19 and 30 analyses per second. Some additional filtering was necessary in order to improve the final data, especially in areas where the surface polish could not be achieved perfectly, resulting in poorer quality diffraction patterns.

### SEM Techniques

SEM microcharacterizations were performed according to Grellet-Tinner [43].

### Ostracod faunas

About 500 g of sediments were collected from the grey marl located just underneath the main limestone level (Figure 3a) in order to determine the association of ostracods, which can provide accurate information on paleoenvironmental and water depth conditions [54]. The samples were washed and sieved using a standard 63 micron sieve. The determination of the ostracod taxa rests on the morphology of the valves, the hinges, and of the muscle scars, based on the classification of Hartman and Puri [55] and Horne et al [56] completed with the works of Meisch [54] and Kempf [57], [58]. *Ilyocypris gibba* lives in warm fresh waters, prefers muddy lake bottoms, and is typically associated with charophyte mats [54]. *Paralynocythere* sp. is typically associated with carbonate muds and, as *Candona cf. spelaea*, prefers oligohaline (0.5–5‰) waters [54], [59], where *Ilyocypris gibba* can also thrive [54]. *Limnocyithere* sp. is typical in low oxygen and alcaline, bicarbonate waters, and can resist high chlorine concentrations [54]. No material was collected from the limestone level where the nest was found due to its indurated nature.

### Organic geochemistry

The organic fraction was isolated, after cleaning the studied material with organic solvents (acetone and dichloromethane) to minimize cross-contamination, following an optimized method developed for extracting organic matter from organic-poor chert samples [60]. Briefly, approximately 200 mg of powdered material was treated in 15 mL of extractant solvent mixture consisted of 60% dichloromethane (HPLC grade, 99.8%) and 40% n-hexane (HPLC grade, 99.8%). The extractable organic matter was isolated using focused ultrasound equipment with a titanium probe (3 mm) during 30 min and applying a sonication power of 50%. Once the extraction procedure was completed, the liquid fraction was separated from the residual solid by centrifugation at 2000 rpm for 15 min. The supernatant was filtered and concentrated to dryness using nitrogen blow-down evaporation at the Turbovap LV Evaporator (Zymark, Hopkinton, MA, USA) and re-dissolved in 100 µL *n*-hexane. The extracted organic fractions were analyzed on a 6890N Agilent gas chromatograph coupled to an Agilent 5973N electron impact ionization mass spectrometer and a 7683 Agilent autosampler. An aliquot of the extract was injected in the splitless mode at 300°C into a HP-5ms (30 m×0.25 mm, 0.25 µm, Agilent) capillary column. The temperature program used for the chromatographic separation was as follows: 60°C (0.5 min), temperature increase at 20°C min^−1^ to 120°C and a second ramp of 6°C min^−1^ up to 300°C, where it was finally held for 15 min. Helium (99.9995%, Carburos Metálicos, Barcelona, Spain) was used as a carrier gas at constant pressure of 13.4 psi. The transfer line temperature was maintained at 310°C, and the ion source and quadrupole at 230°C and 150°C respectively. The analyses were performed in SCAN mode in order to detect all the organic compounds present in the target materials. Paleoenvironment conditions were inferred from the distribution patterns of n-alkanes alone, as the concentration of hopanoid compounds were close to the limit of detection.

### Strontium isotopes

Strontium isotopic data of eggshell from the avian nest and its embedding limestone were analyzed in order to characterize the water paleo-lake chemistry. Studied samples (three eggshell fragments and two neighboring limestone fragments) were carefully cleaned using an ultrasound bath. About 5 mg of samples were transferred to Teflon beakers and digested in 2 ml of 2N HNO3 (analytical-reagent grade, purified by performing sub-boiling distillation in silica glass stills). Samples were then loaded into cation exchange columns packed with Sr.spec® strontium-selective resin to isolate strontium from other ions. Sr was loaded onto a double Re-Ta assembly of outgassed filaments and measured on a Finnigan MAT 262 thermal ionisation mass spectrometer at the University of the Basque Country (Spain). Multiple samples of the strontium standard NBS-987 were run to confirm instrument accuracy. External precision of analysis was ±0.00002 (2 sigma absolute) based on 206 analyses of NBS-987. Replicate analysis of the NBS 987 Sr standard during runs gave ^87^Sr/^86^Sr 0.710281±12 (2s, n = 2).

## Supporting Information

File S1 **Vs-1 BSEM AND EDS.** (a) BSEM microcharacterization neatly shows Vs-1 three structural layers but also the thin outermost covering (black arrows) typical of the Podicipediformes+Phoenicopteriformes clade. (b) EDS analysis reveals the original covering has been replaced during fossilization by Mg, Na, Al, Si and Ca, elements represented in high concentrations in the Bardenas paleo-endorheic lake.
(TIF)

File S2 **Eggshell SEM comparisons.** (a) Vs-1; (b) White Ibis (*Eudocimus albus*); (c) Spoonbill (*Platalea ajaja*); (d) Great Bustard (*Otis tarda*); (e) American Flamingo (*Phoenicopterus ruber*); (f) White Stork (*Ciconia ciconia*); (g) BSEM of Vs-1; (h) Western Grebe (*Aechmophorus occidentalis*). Note the extreme similarities between (a) and (e) and the contrasting differences between (a) and the other eggshells even (h) a grebe, the flamingo sister taxon. Vs-1 BSEM (g), confirms that the SEM image of Vs-1 is not biased by microscopic artefact and the resemblance between the fossil and modern flamingo species (e) is not coincidental. Although minor differences in the proportions of the eggshell units are discernable between (a) and (e) their overall congruence supports a close relationship.
(TIF)

File S3 **Nests, nesting sites, and eggs of modern flamingos and grebes.** (a) Breeding colony of captive flamingos. Note the volcano-shaped nest with one single egg each, and the extreme shallow lacustrine environment. (b) Andean punas in Catamarca (Argentina). The white in the back and fore ground is salt deposition from small hydrothermal activities in shallow endorheic lakes. Yet, glacier melt water contributions increase episodically this lake level and conversely decrease the salt concentration. Hyperhaline conditions seem to be the dominant factor that favours flamingo nesting rather than temperatures. (c) and (d), flamingo and grebe eggs respectively. Note the substantial size difference between the eggs of these 2 species that are sister taxa. Vs-1 matches perfectly the egg of modern grebes.
(TIF)
