# Supplementary figures and images for: The First Occurrence in the Fossil Record of an Aquatic Avian Twig-Nest with Phoenicopteriformes Eggs: Evolutionary Implications
Source: PLoS One. 2012 Oct 17;7(10):e46972. doi: 10.1371/journal.pone.0046972 (PMC3474766; doi:10.1371/journal.pone.0046972)

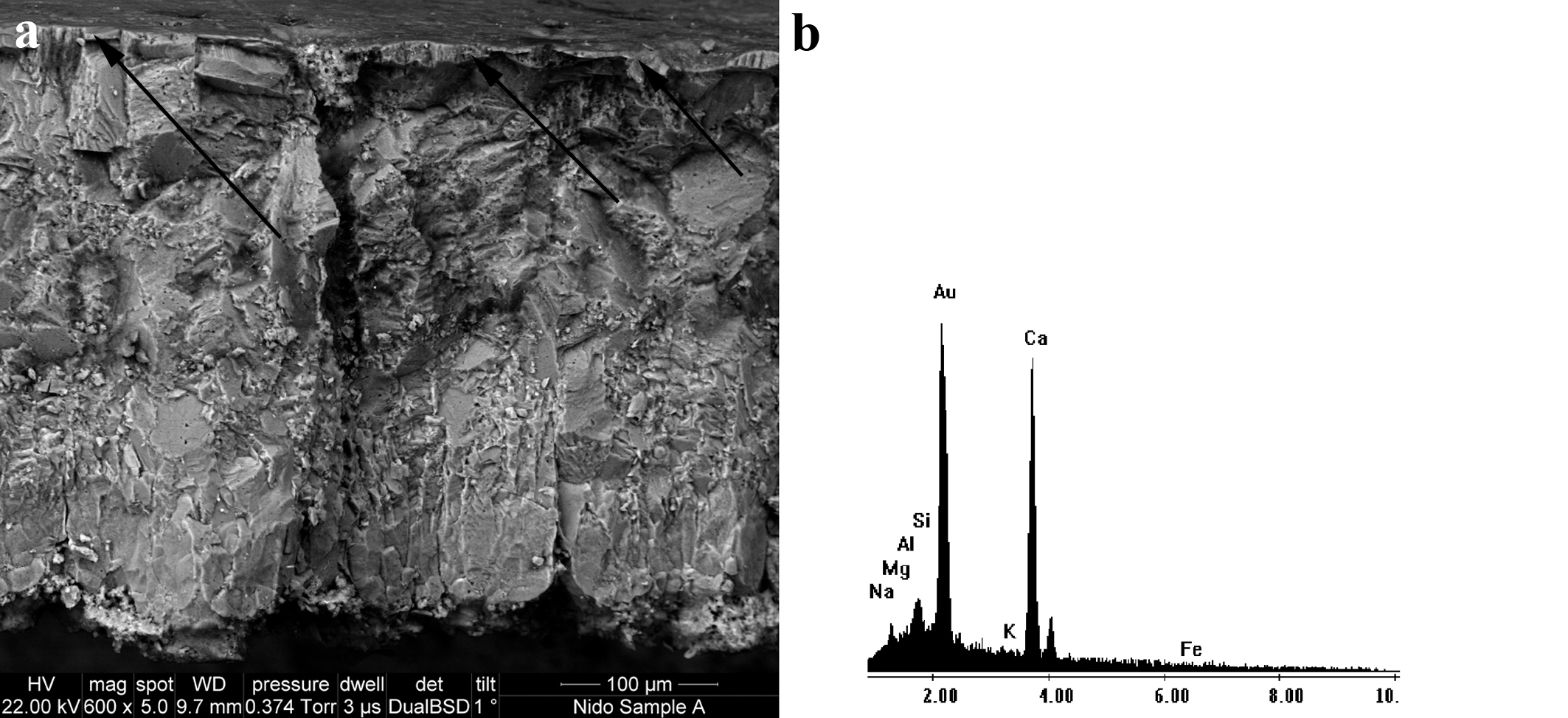

Supplement: File S1 — Vs-1 BSEM AND EDS. (a) BSEM microcharacterization neatly shows Vs-1 three structural layers but also the thin outermost covering (black arrows) typical of the Podicipediformes+Phoenicopteriformes clade. (b) EDS analysis reveals the original covering has been replaced during fossilization by Mg, Na, Al, Si and Ca, elements represented in high concentrations in the Bardenas paleo-endorheic lake. (TIF) [file pone.0046972.s001.tif]

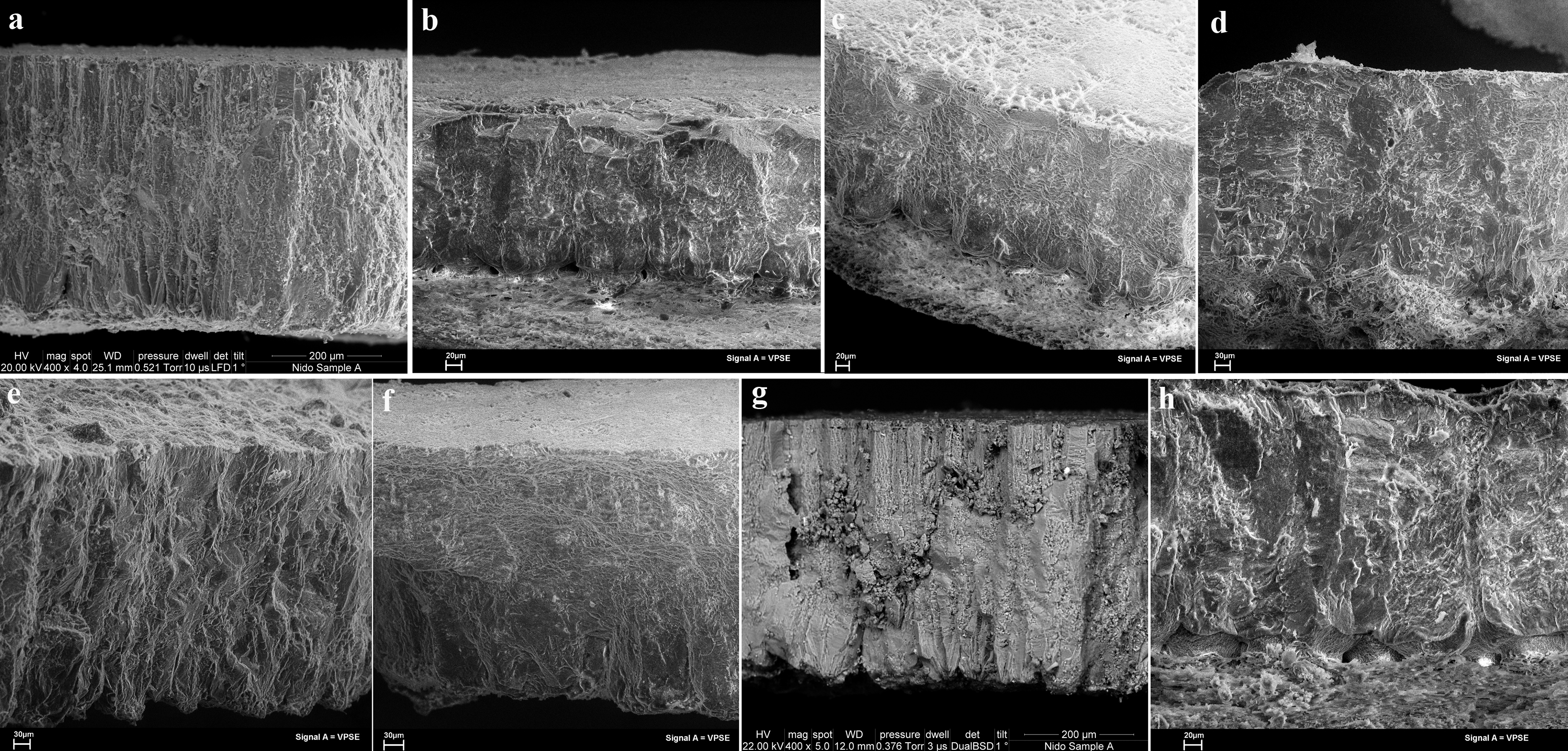

Supplement: File S2 — Eggshell SEM comparisons. (a) Vs-1; (b) White Ibis (Eudocimus albus); (c) Spoonbill (Platalea ajaja); (d) Great Bustard (Otis tarda); (e) American Flamingo (Phoenicopterus ruber); (f) White Stork (Ciconia ciconia); (g) BSEM of Vs-1; (h) Western Grebe (Aechmophorus occidentalis). Note the extreme similarities between (a) and (e) and the contrasting differences between (a) and the other eggshells even (h) a grebe, the flamingo sister taxon. Vs-1 BSEM (g), confirms that the SEM image of Vs-1 is not biased by microscopic artefact and the resemblance between the fossil and modern flamingo species (e) is not coincidental. Although minor differences in the proportions of the eggshell units are discernable between (a) and (e) their overall congruence supports a close relationship. (TIF) [file pone.0046972.s002.tif]

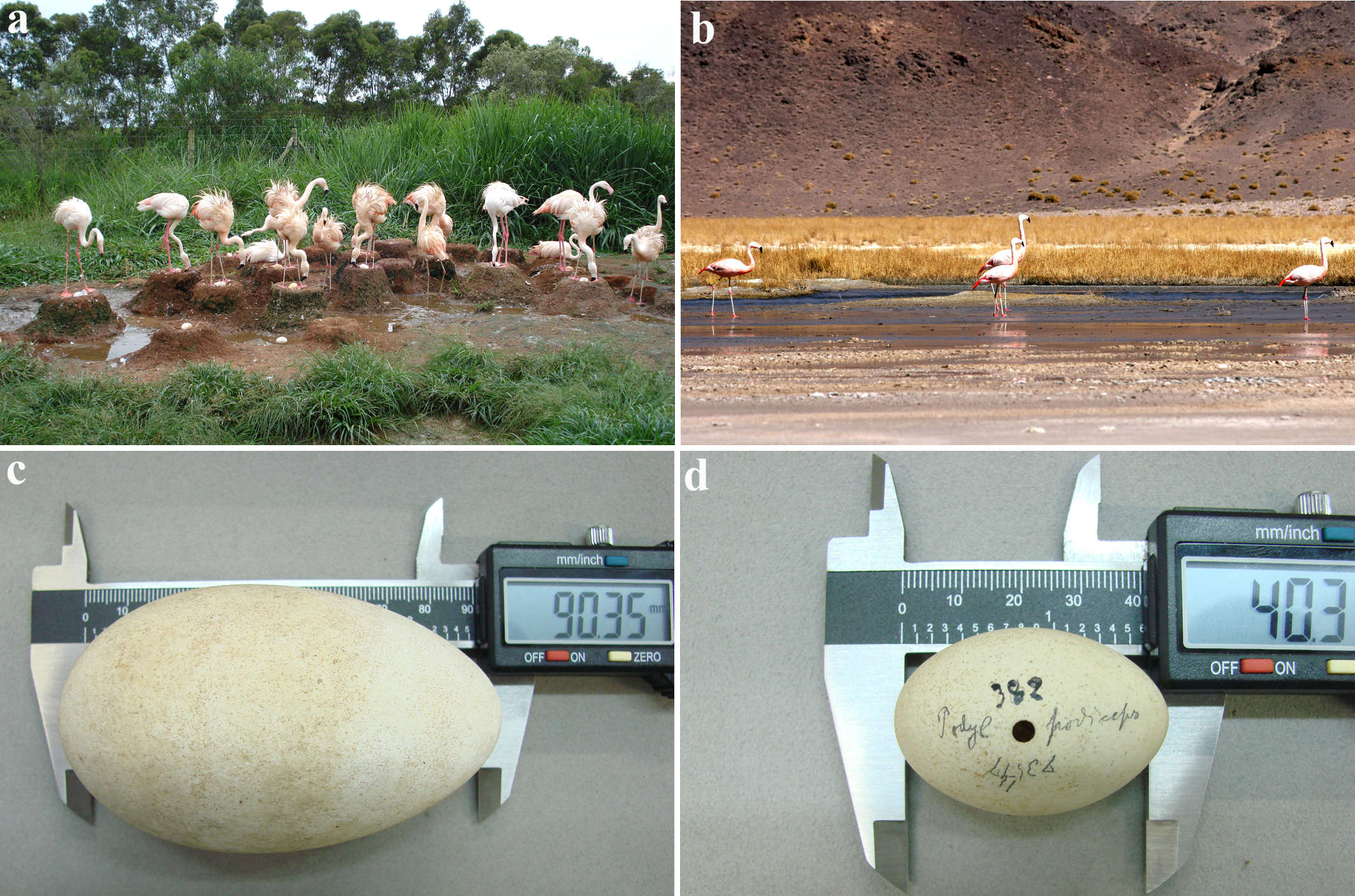

Supplement: File S3 — Nests, nesting sites, and eggs of modern flamingos and grebes. (a) Breeding colony of captive flamingos. Note the volcano-shaped nest with one single egg each, and the extreme shallow lacustrine environment. (b) Andean punas in Catamarca (Argentina). The white in the back and fore ground is salt deposition from small hydrothermal activities in shallow endorheic lakes. Yet, glacier melt water contributions increase episodically this lake level and conversely decrease the salt concentration. Hyperhaline conditions seem to be the dominant factor that favours flamingo nesting rather than temperatures. (c) and (d), flamingo and grebe eggs respectively. Note the substantial size difference between the eggs of these 2 species that are sister taxa. Vs-1 matches perfectly the egg of modern grebes. (TIF) [file pone.0046972.s003.tif]
